# Utilization of Skilled Birth Attendance among Mothers Who Gave Birth in the Last 12 Months in Kembata Tembaro Zone

**DOI:** 10.1155/2022/8180387

**Published:** 2022-08-30

**Authors:** Eyassu Mathewos Oridanigo, Belete Kassa

**Affiliations:** ^1^Department of Nursing, College of Medical and Health Sciences, Wachemo University, Durame Campus, Durame, Ethiopia; ^2^Angacha Woreda Health Office, Kembata Tembaro Zone, P.O. Box 20, Angacha, Ethiopia

## Abstract

**Background:**

Skilled delivery is considered the single most important strategy in preventing maternal and neonatal morbidity and mortality. It ensures safe birth, reduces actual and potential complications, and increases the survival of most mothers and newborns.

**Objective:**

To identify determinants of the utilization of skilled birth attendance among women who gave birth in the last 12 months in the Kembata Tembaro zone, Southern Ethiopia, 2020.

**Methods:**

A community-based cross-sectional study was employed from 1 April 2020 to 30 April 2020 among women who gave birth in the last 12 months in the Kembata Tembaro zone. Six hundred twenty-four mothers were recruited for the study as eligible participants. Multistage stratified sampling was used to select three districts and one town administrative unit of the study area. The data were collected and verified for their completeness, followed by editing and coding. Multivariate analysis was performed using the backward LR method to identify factors independently associated with the dependent variable. Statistical significance was declared at a *p* value of less than 0.05, and the strength of statistical association was measured by adjusted odds ratio and 95% confidence interval.

**Result:**

Of 624 study subjects sampled, 607 provided information with a response rate of 97.3%. In this study, 309 (50.9%) women had their last birth at health facilities attended by skilled birth attendants. Place of residence (AOR (95% CI) = 0.33 (0.22,0.58)); age at interview (AOR (95% CI = 3.41 (1.57,5.45)); maternal education (AOR (95% CI) = 1.50 (1.34, 4.19)); history of still birth (AOR (95% CI) = 3.85 (2.14,6.91)); maternal occupation (AOR (95% CI) = 3.35 (1.79,6.27)); husband occupation (AOR (95% CI) = 2.69 (1.70,7.09)); ANC visit (AOR (95% CI) = 4.62 (3.12,7.32)); knowledge of obstetric complications (AOR (95% CI) = 3.10 (1.37,5.21)); and final decision-making about place of delivery (AOR (95% CI) = 3.64 (1.70,7.99)) were significantly associated with the use of skilled birth attendance.

**Conclusion:**

In this study, nearly half of the mothers used skilled birth attendance. Place of residence, age at interview, maternal education, history of still birth, maternal occupation, husband occupation, antenatal visit, knowledge about obstetric complications, and final decision-maker about place of delivery were determinants of the use of skilled attendance delivery.

## 1. Background

According to the World Health Organization (WHO), “skilled birth attendants are accredited health professionals (such as midwives, doctors, or nurses) who have been educated and trained to proficiently manage normal (i.e., uncomplicated) pregnancies, childbirths and the immediate postnatal period, as well as handle the identification, management, and referral of complications in women and newborns” [1]. To reduce maternal mortality, the indicators of progress are a proportion of births attended by skilled attendants and the maternal mortality ratio (MMR) [2]. Skilled birth attendance (SBA) during labor, delivery, and early postpartum period can significantly reduce both maternal and newborn morbidity and mortality by preventing or managing most obstetric complications [3]. Providing skilled care at birth goes hand in hand with the sustainable development goals (SDGs) to reduce child mortality, particularly neonatal mortality [4]. Since 2000, the United Nations' Millennium Development Goals (MDGs), which included a goal to improve maternal health by the end of 2015, have facilitated significant reductions in maternal morbidity and mortality worldwide [5]. Despite more focused efforts made especially by low- and middle-income countries, targets were largely unmet in sub-Saharan Africa, where women are plagued by many challenges in seeking obstetric care [6].

Maternal mortality is unacceptably high, and every day approximately 810 women are died from preventable causes related to pregnancy and childbirth in 2017 [6]. Sub-Saharan Africa and Southern Asia accounted for approximately 86% (254 000) of the estimated global maternal deaths in 2017 with sub-Saharan Africa alone accounting for roughly 66% (196 000) deaths [7]. Nearly 42.5% of infant deaths each year occur within the first week of life and are often due to a lack of or inappropriate care during pregnancy, delivery, and the post-partum period [8]. One-third of nearly one million stillbirths occur during labor, and approximately 280,000 babies die of birth asphyxia soon after birth. Approximately 60% of African women and their babies do not receive skilled care during childbirth, and fewer receive effective postnatal care [9]. This is also the crucial time for other interventions, especially the prevention of mother-to-child transmission of HIV and initiation of breastfeeding [10].

In Ethiopia, poor access to SBA is reflected by its MMR [11]. According to 2016 Ethiopian Demographic and Health Survey (EDHS), MMR was estimated to be 412 per 100,000 live births [12]. Major causes of maternal deaths in Ethiopia are like to most developing countries such as infection, hemorrhage, obstructed labor, abortion, and hypertension that could be avoided if preventive measures were taken and adequate care is available particularly during pregnancy, childbirth, and postpartum period through obstetric care services [13]. Poor access to and use of skilled delivery services have been identified as a major contributory factor to high maternal and newborn mortality, which remains a major challenge to health systems and public health issues in the country [14].

Although skilled delivery has been promoted in Ethiopia, home delivery with traditional birth attendants (TBAs) is still common, primarily in rural areas that are hard to reach [15]. The 2016 EDHS showed that only 28% of live births in the 5 years before the survey were delivered by a skilled provider, 26% in the health facility, whereas home delivery was 73% and 1% in other places. For rural women, the report showed that 80 percent of births to urban mothers were assisted by a skilled provider as compared to 21 percent in the rural areas. 80% of them gave birth at home [16].

Based on the National Reproductive Health Strategy (NRHS), the country planned to increase the proportion of births attended by skilled health personnel either at homes or in the facilities to 60% [5]. Despite the efforts being made by the government and other stakeholders to mitigate the problems and subsequent consequences posed by SBA delivery, studies in different parts of the country are showing that most Ethiopian women are giving birth at home and SBA remains low [17, 18]. To enhance use of SBA in the country, barriers during delivery among women need to be identified across the regions. This research can lead to an improvement in the clinical practice of institutional delivery with SBA in Africa including Ethiopia where 90% of births take place at home with traditional birth attendants (TBAs) and the proportion of deaths due to postpartum hemorrhage that may threaten the survival of the mother. Little is known about the current magnitude of use of SBA and its determinants in the study area. Therefore; this study aimed at assessing the extent of SBA utilization and attempted to explore its determinants that are assumed to be barriers among mothers who gave birth in the last 24 months in the Kembata Tembaro zone, Southern Ethiopia.

## 2. Methodology

### 2.1. Study Area and Period

A community-based cross-sectional study was conducted in the Kembata Tembaro zone from April 1 to 30, 2020. The zone is located in the Southern Nations, Nationalities and People Republic (SNNPR) of Ethiopia and its capital town, Durame, which is located 293 kilometers (km) south of Addis Ababa and 118 km west of Hawassa. In this zone, there are eight woreda health offices and four health administrative health units, one general and four primary hospitals, 28 governmental and three non-governmental health centres, 136 health posts, and 1170 different types of health professionals.

### 2.2. Population

The source population consisted of all women who gave birth in the last 12 months before the survey in the study area, while the study population consisted of randomly selected women who gave birth in the last 12 months, irrespective of the outcome of the birth.

### 2.3. Sample Size Determination

To determine the sample size, two-population proportion formulas were used, and the following assumptions were made. The level of confidence = 95% and power = 80%. Antenatal Care (ANC) visit during last the pregnancy was considered a factor for the utilization of SBAs. Participants were categorized as women who visited or did not visit ANC during last their pregnancy [19]. An ANC visit during the last pregnancy gives the maximum sample size among other predictor variables, such as a place of residence and educational status.

*P*1 = Proportion of women who attended ANC during their last pregnancy = 57.9%

*P*2 = Proportion of women who did not attend ANC during their last pregnancy = 42.1%

Based on the above assumptions, a design effect of 1.5 and a 5% nonresponse rate, 624 study participants who gave birth in the last 24 months were selected for the study.

### 2.4. Sampling Procedures

Multistage stratified sampling was used to select three districts (Angacha, Doyogena, and Kedida Gamela) and one administrative town, Durame, from a total of eight districts and four administrative towns in the zone. First, the zone was stratified into rural districts and urban administrative towns, and then, 15 kebeles were chosen by lottery. House-to-house visits were carried out in selected kebeles to identify households with women who gave birth in the last 12 months prior to the survey, and 7806 households were identified as fulfilling the eligibility criteria. By allocating the sample size proportionally to each kebele, systematic sampling was used to select study subjects. If the houses were closed or the mother was not present at the time of data collection, revisits were made until the data collectors were able to survey the women.

### 2.5. Variables

#### 2.5.1. Dependent Variable: Use of Skilled Birth Attendance

Independent variables are as follows: socioeconomic and demographic factors (education, age, marital status, education income, number of family members, residence, travel time to the nearest health facility within 30 minutes, exposure to the mass media), obstetric factors, parity, complications experienced (prolonged labor), history of stillbirth, history of ANC follow-up, husband's factors (occupation, education), and knowledge and attitude on key danger signs of pregnancy, labor/childbirth, and delivery services.

### 2.6. Data Collection Procedures

Data were collected using interviewer-administered, structured questionnaires that were developed after reviewing relevant studies [17, 19–22]. Six BSc nurses and one health officer were recruited to collect the data and supervise the data collection process, respectively. Data collectors were selected from outside the study area to minimize interviewer bias and selected based on the ability to speak both Kambatissa and Amharic (local languages). Two days of training were provided concerning the purpose of the study and the methods of data collection. The supervisors were informed about the strict supervision and cross-checking procedures for data abstraction forms and completeness at the end of each day. The principal investigator supervised the overall activities.

### 2.7. Data Quality Control

The quality of the data was assured via proper questionnaire design and training of data collectors and supervisors for two days before the data collection. Every day after the data collection, the questionnaires were reviewed and checked by supervisors to maintain accuracy and completeness. The English versions of the questionnaires were translated into local languages (Kambatissa and Amharic) and back-translated to English, and comparisons were made to ensure the consistency of these versions. Data collection tools were pretested at 5% of the sample to identify any weaknesses in the structuring of the research instruments prior to their use in data collection. Following the pretest, the tools were improved in terms of their clarity and simplicity.

### 2.8. Data Management and Statistical Analysis

Data were checked for its completeness, and edited, coded, and cleaned; and then were entered into EpiData version 3.1 and exported to SPSS version 23 for analysis. Descriptive statistics were computed, and the results were presented by tables and graphs, and the numerical summary was used to present the results. Before bivariate analysis, all variables were checked by cross-tabulation for fulfilling chi-squared test assumptions of 80% expected frequency greater than five and all cells expected frequency greater than one. Variables with *p* < 0.25 in bivariate analysis were considered candidates for multivariate analysis. Multivariate analysis was performed using the backward LR method to identify factors independently associated with the dependent variable. Statistical significance was declared with *p* < 0.05, and the strength of statistical association was measured by adjusted odds ratio and 95% confidence intervals. Hosmer–Lemeshow goodness-of-fit statistics were used to check the goodness of fit of the model with a *p*-value of 10%.

### 2.9. Operational Definitions

#### 2.9.1. Utilization of Skilled Birth Attendance

Use of skilled birth attendance delivery was assessed by asking the mother if she gave birth only in hospital or health centre assisted with healthcare providers with midwifery skills or not for her recent delivery within the last 12 months.

#### 2.9.2. Knowledgeable on Danger Signs of Pregnancy

A woman was considered knowledgeable if she could mention at least three danger signs that could occur during pregnancy [17, 20].

#### 2.9.3. Knowledgeable on Danger Signs of Labor/Childbirth

A woman was considered knowledgeable if she could mention at least three danger signs that could occur during labor/childbirth and not knowledgeable if otherwise [17, 20].

#### 2.9.4. Knowledgeable on Key Danger Signs of Postpartum

A woman was considered knowledgeable if she could mention at least the three danger signs that could occur during postpartum period/after delivery and not knowledgeable if otherwise [17, 20].

## 3. Results

### 3.1. Sociodemographic and Socioeconomic Characteristics of the Respondents

In this study, out of 624 participants sampled, 607 of them provided information with a response rate of 97.3%. Approximately two-third of the study subjects, 395(65.1%) were in the age range of 25–34 years with a mean and standard deviation age of 27.3 and 5.6, respectively, and 479(68.6%) were residing in the rural areas. The majority of the respondents, 446 (73.5%), were Kembata in ethnicity. Regarding the educational level of respondents, nearly half, 306 (50.5%), attended secondary and above schools (Table 1).

### 3.2. Obstetric Characteristics of the Respondents

**Figure 1:**
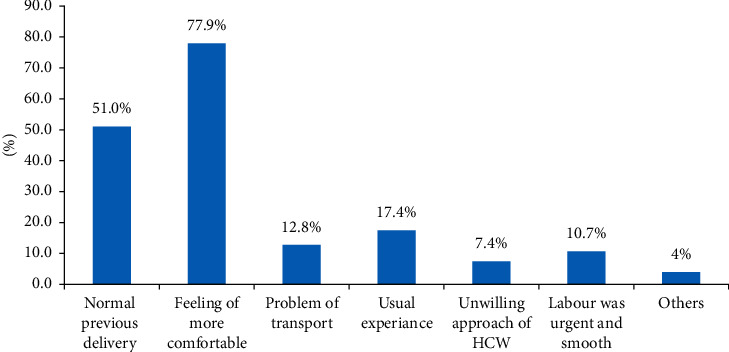
The reasons for home delivery among the respondents in the Kembata Tembaro zone, Southern Ethiopia.

Among the respondents, 187 (30.8%) (rural: 133 (21.9%) and urban: 54 (8.9%)) married before the age of 18 years. Regarding age at the first pregnancy, 181 (29.8%) respondents (rural: 145 (23.9%) and urban: 36 (5.9%)) were pregnant before the age of 20. More than half, 354 (58.3%) did not have ANC follow-up during their last pregnancy, and among those who had ANC follow-up history, only 192 (31.6%) had four visits and above. Among the respondents, nearly half, 232 (49.1%), reported that they gave their last birth at home and more than half, 168 (56.4%), were attended by TBAs (Table 2). Regarding the reason for home delivery, nearly three-fourth, 232 (77.9%), of the respondents reported that the main reason for home delivery was feeling more comfort (Figure 1).

### 3.3. Accessibility Characteristics of Respondents

Approximately half of respondents, 305 (50.2%) had health facility within 1 to 2 hours distance, while 220 (36.2%) and 82 (13.5%) had health facilities within one hour and less than one hour distance, respectively. Regarding availability of functional media, 351 (57.8%) and 109 (18%) had functional media (radio and/or television), but 147 (24.2%) had no functional media at all.

### 3.4. Knowledge on Key Obstetric Danger Signs during Pregnancy, Labor, and Childbirth, and After Delivery

In this study, 289 (47.6%), 498 (82.0%), 326 (53.7%), 252 (41.5%), 208 (34.3%), and 310 (51.1%) mentioned severe headache, blurred vision, vaginal bleeding, severe abdominal pain, loss of consciousness, and convulsion during pregnancy, respectively. Regarding danger signs during labor and childbirth, 421 (69.4%), 539 (88.8%), 559 (92.1%), 425 (70%), and 369 (60.8%) mentioned severe vaginal bleeding, prolonged labor, retained placenta, loss of consciousness, and convulsion, respectively. Moreover, 538 (88.6%), 460 (75.8%), 424 (69.9%), 325 (53.5%), and 356 (58.6%) mentioned retained placenta, excessive bleeding, abdominal pain, vaginal discharge, and severe headache, respectively. Based on the above signs, about half, 315 (51.9%) respondents were knowledgeable on obstetric complications related to labor and childbirth (Table 3).

### 3.5. Women's, Husbands', and Family-Related Factors

Regarding decision on the place of delivery, about two-fifth of the respondents, 256 (42.2%), reported that the decision was made by themselves (urban: 75 (39.3) and rural: 181 (43.5)). Regarding mothers' preferences about place and attendant of delivery, more than half, 347 (57.2%) and 279 (46%), preferred home delivery and SBA, respectively (Table 4).

### 3.6. Utilization of Skilled Birth Attendance Delivery

**Figure 2:**
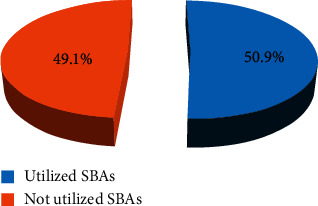
Utilization of SBA of respondents in the Kembata Tembaro zone, 2020.

In this study, 309 (50.9%) women gave their last birth at health institutions being attended by skilled birth attendants (urban: 134 (43.4%) and rural: 175 (56.6%)) (Figure 2).

### 3.7. Factors Affecting Skilled Birth Attendance Utilization

Among the variables in bivariate analysis, 14 of them had a *p*-value of less than 0.25; hence, they were candidates for multivariate analysis. They were again entered into multiple logistic regression models to obtain variables that were independently associated with the use of skilled birth attendance. The variables with a *p*-value of less than 0.05 in multivariate analysis were taken as significant predictors of outcome variable.

Therefore, the final model showed that there was statistically significant association between ANC follow-up and utilization of SBA delivery (*p*-value< 0.001) so that mothers who had at least four ANC visits were 4.62 times more likely to use skilled birth attendance than those who had less than four ANC visits during their last pregnancy (OR (95% CI) = 4.62(3.12, 7.32)). In this study, we found that there was negative association between the place of residence and utilization of skilled birth attendance (*p* < 0.001). Mothers who lived in rural areas were 67% less likely to use skilled birth attendance than those who lived in urban areas (OR (95% CI) = 0.33(0.22, 0.58)) (Table 5).

## 4. Discussion

Delivery assisted by skilled providers is the most proven intervention in reducing maternal mortality and one of the targets of SDGs of the United Nations (UN) [23]. This community-based study identified very important determinants that are related to SBA utilization among respondents. The findings of the study revealed that the proportion of women who delivered in the facility assisted by skilled birth attendants was 51.8%. This finding is higher than studies conducted in different parts of Ethiopia [17, 19, 24, 25]. This might be because of the functions of multipurpose health extension workers on improvements in ANC follow-up and facilitating referral services to HCs and hospitals for delivery service assisted by skilled healthcare providers. Health extension workers improved the utilization of maternal health services including skilled birth attendance delivery by bridging the gap between communities and health facilities [26]. However, it was lower than the study conducted in rural southern Ghana where 68.8% of mothers were assisted by skilled providers during their last delivery [21]. The difference could be explained by the fact that women in those countries had better socioeconomic status.

In this study, the place of residence was statistically significant and negatively associated with the utilization of SBA. The result showed that mothers who lived in the rural areas were less likely to use SBA than those who lived in the urban areas. This finding is supported by studies conducted in different regions of the country [17, 19, 22, 25, 27–29]. The possible reason might be prevailing traditional thinking/views, presence of low education and income, lack of awareness on maternal health services like ANC, birth preparedness and complication readiness, and remoteness/lack of transportation to the health facility for mothers in rural than urban areas [30]. History of still birth was another predictor of utilization of SBA. This study revealed that mothers who had previous history of still birth were more likely to use SBA than mothers who did not have still birth. The finding from a cross-sectional survey conducted in the Dembecha district of Northwest Ethiopia showed the negative association [24]. The possible reason might be the fact that ladies who had still birth in their lifetime may have a fear to develop complications during the delivery of their child and prefer skilled providers to give birth in the health facilities.

Older women were more likely to give birth assisted by skilled birth attendants than young women. This finding is similar to the study performed in rural residents of Southern Ghana [21]. However, the finding opposes other studies conducted in the Raya district of North Ethiopia and Ghana, which found young women were more likely to use SBA than older women [19, 31]. This might be because older women were able to consider that giving birth at home is risky as they had experienced previously and they might get additional information regarding risk of home delivery with TBAs during different visits (childcare, immunization services, etc.) to health facilities. The higher age of women can influence their status in society, which has been found to increase the ability of decisions [32].

Mothers' educational status was another predictor of the utilization of skilled birth attendants, which was statistically significant. Mothers who could read and write as well as mothers who learned secondary and above were more likely to use SBA than those who were unable to read and write. This finding is consistent with the report from EDHS 2016, which found a strong correlation between mothers' educational status and skilled birth attendant delivery. EDHS 2016 found that 17% of births to mothers with no education were assisted by a skilled provider as compared to 93% and 92% of births to mothers with more than secondary education, respectively [16]. This might be because educated women are likely to make their own healthcare decisions more and seek proper health care than their counterparts. In this study, parity was negatively associated with SBA utilization.

Maternal occupation is an important predictor of the use of SBA. The study showed that both the government employees and merchants were more likely to use SBA than housewives. It was supported by the study conducted in Northern Ethiopia and rural areas of southern Ghana, which showed an important association between the occupational status of mothers and the use of SBA delivery [19, 21]. Mothers with government-employed husbands were also more likely to utilize SBA delivery than farmers. This finding was supported by the study performed in the Gamo Gofa zone, southern Ethiopia [20]. The possible reason might be because those government employees and merchant ladies and their husbands might have more income and awareness for identifying skilled providers and places of delivery, searching for money for incurred costs, finding transportation, and other things, which may contribute to home delivery.

In this study, we found that ANC visit during the last pregnancy of the respondents was significant with use of SBA. Women who had ANC visits greater than or equal to four times with skilled professionals during their last pregnancy were more likely to use SBA than those who had less than four visits. This finding was also supported by the report from EDHS of 2011 and other studies conducted in different parts of Ethiopia [17, 19, 27, 33]. This might be women during ANC follow-up can obtain counseling services on birth preparedness including the place of delivery and selection of birth attendant and complication readiness. ANC from a skilled provider is important to monitor pregnancy and reduce morbidity and mortality risks for both the mother and child during pregnancy, delivery, and the postnatal period so that those mothers who had a history of ANC follow-up can easily give attention to deliver in the HF with SBA [16].

Knowledge regarding health problems during pregnancy and childbirth was another important predictor of the use of SBA. Those respondents who knew danger signs of pregnancy and childbirth were more likely to utilize SBA than those who did not know them. It is consistent with studies conducted in the Raya district of North Ethiopia and Gura Dhamole Woreda, Bale zone, southeast Ethiopia [17, 19]. Women can take action by seeking appropriate health care by recognizing danger signs during pregnancy, which can help them to deliver in the health facility with skilled birth attendants [34].

Moreover, the final decision-maker about the place of delivery in the last pregnancy was another important predictor, which is significantly associated with the use of SBA. Respondents who jointly (both wife and husband) decided were more likely to use SBA than respondents who decided by themselves about the place of delivery. This finding is supported with different studies conducted [17, 19, 24, 27, 35]. If women are encouraged by their husbands, they would also get financial and other social support to go to health facilities, which will allow them to have health provider-assisted delivery [36]. In contrast to this, studies conducted in western Ethiopia have shown that women whose decision was made by themselves were two times more likely to utilize SBA than women whose decision was made by others on the place of delivery [37].

### 4.1. Limitation

Due to the cross-sectional study design, no causal inferences can be made regarding the temporal association between the potential factors and utilization of SBA.

## 5. Conclusion

In this study, about half of the study subjects were utilizing SBA. Women's place of residence was negatively associated, while maternal education, maternal occupation, husband occupation, age at interview, ANC visit, knowledge about obstetric complications, during and after child birth, final decision-maker about place of delivery, and history of still birth were positively associated with outcome variable and utilization of SBA.

## Figures and Tables

**Table 1:** Sociodemographic and socioeconomic characteristics of respondents in the Kembata Tembaro zone, 2020.

Variables (*n* = 607)	Category of characteristics	ANC follow-up					
		Yes		No		Total	
		*N*	%	*N*	%	*N*	%
Age categories in years	15–24	57	9.4	81	13.3	138	22.7
	25–34	252	41.5	143	23.6	395	65.1
	35 and above	34	5.6	40	6.6	74	12.2

Mother's educational status	Unable to read and write	5	0.8	22	3.6	27	4.4
	Read and write only	89	14.7	185	30.5	274	45.1
	Secondary and above	249	41	57	9.4	306	50.5

Husband's educational status	Unable to read and write	7	1.2	15	2.5	22	3.6
	Read and write only	75	12.4	142	23.4	217	35.8
	Secondary and above	261	42.9	107	17.6	368	60.6

Residence	Urban	121	19.9	70	11.5	191	31.5
	Rural	222	36.6	194	32	416	68.5

Mother's occupational status	Housewife	175	28.8	197	32.5	372	61.3
	Merchant	118	19.5	50	8.2	168	27.8
	Employee (government/private)	50	8.2	17	2.8	67	10.9

Husband occupational status	Farmer	199	32.9	154	25.4	353	58.2
	Employee (government/private)	48	7.9	25	4.1	73	12
	Merchant	80	13.2	75	12.4	155	25.5
	Daily laborer	16	2.6	10	1.6	26	4.3

Ethnicity	Kembata	254	41.8	191	31.5	446	73.5
	Amhara	38	6.3	31	5.1	69	11.4
	Guraghe	32	5.3	20	3.3	52	8.6
	Others (^#^)	19	3.1	21	3.5	40	6.6

Religion	Protestant	298	49.1	206	33.9	504	83.1
	Orthodox	23	3.8	35	5.8	58	9.5
	Muslim	15	2.5	17	2.8	32	5.3
	Others (^*∗*^)	7	1.2	6	1	13	2.1

Exposure to media	Radio	229	37.7	122	20.1	351	57.8
	Television	77	12.7	32	5.3	109	18.0
	Not exposed	37	6.1	110	18.1	147	24.2

Parents' monthly income in ETB	Below 500	194	32.0	213	35.1	407	67.1
	501–999	91	15.0	36	5.9	127	20.9
	1000–1499	45	7.4	8	1.3	53	8.7
	≥1500	13	2.1	7	1.2	20	3.3

Number of family members	One	7	1.2	6	1	13	2.1
	Two	20	3.3	12	2	32	5.3
	Three	83	16.7	32	5.3	115	18.9
	Four	90	14.8	41	6.8	131	21.6
	More than four	143	23.6	173	28.5	316	52.1

*Note.* Others (^#^) indicate Oromo, Tigre, Hadiya, and Wolaita ethnicities, and others (^*∗*^) indicate Adventist, Hawarat, and Catholic religion followers.

**Table 2:** Last obstetric characteristics of respondents by residential area, Kembata Tembaro zone, Southern Ethiopia, 2020.

Variables (*n* = 607)	Category of characteristics	Rural		Urban		Total	
		*N*	%	*N*	%	*N*	%
Age at first marriage	<18 years	133	21.9	54	8.9	187	30.8
	≥18 years	283	46.6	137	22.6	420	69.2

Age at first pregnancy	<20 years	145	23.9	36	5.9	181	29.8
	≥20	271	44.6	155	25.5	426	70.2

Gravidity	1	100	16.5	21	3.5	121	19.9
	2–4	120	19.8	103	17	223	36.7
	≥5	196	32.3	67	11	263	43.3

Parity	1	117	19.3	34	5.6	151	24.9
	2–4	68	11.2	133	21.9	201	33.1
	≥5	231	38.1	24	3.9	255	42

History of abortion	Yes	47	7.7	18	3	65	10.7
	No	369	60.8	173	28.5	542	89.3

History of still birth	Yes	47	7.7	6	1	53	2.1
	No	409	67.4	185	30.5	554	97.9

Last pregnancy planned	Yes	102	16.8	99	16.3	201	33.1
	No	314	51.7	92	15.2	406	66.9

Birth preparation	Yes	83	13.7	132	21.7	215	35.4
	No	333	54.9	59	9.7	392	64.6

ANC visit during last pregnancy	Yes	271	44.6	154	25.4	425	70.0
	No	102	16.8	80	13.2	182	30.0

ANC frequency (n = 198)	<4 visit	141	23.2	92	15.2	233	38.4
	≥4 visit	90	14.8	102	16.8	192	31.6

Place of delivery within the last 24 months	Health facility	175	56.6	134	43.4	309	50.9
	Home	215	35.4	37	6.1	298	49.1

Assistance during home delivery	My mother	19	3.1	10	1.6	49	16.4
	TBA	151	24.9	17	9.4	168	56.4
	Other family members	45	7.4	10	1.6	81	27.2

Duration of last labor	<12 hrs	98	16.1	58	9.6	156	25.7
	12–24 hrs	211	34.8	108	17.8	329	54.2
	>24 hrs	107	17.6	25	4.1	122	20.1

PNC visit after last delivery	Yes	64	10.5	98	16.1	401	66.1
	No	352	58	93	15.3	206	33.9

Experience adverse Pregnancy outcome	Yes	73	12	35	5.8	108	17.8
	No	343	56.5	156	25.7	499	82.2

**Table 3:** Knowledge status of respondents on key obstetric danger signs during pregnancy, labor, and childbirth and after delivery in the Kembata Tembaro zone, 2020.

Variables (*n* = 607)	Category	Frequency	Percent
Knowledge on danger signs related to pregnancy	Knowledgeable	261	43.0
	Not knowledgeable	195	32.1

Knowledge on obstetric complications related to labor and childbirth	Knowledgeable	315	51.9
	Not knowledgeable	292	48.1

Knowledgeable on danger signs related to postpartum	Knowledgeable	287	47.3
	Not knowledgeable	320	52.7

**Table 4:** Preferences of the respondents, their husbands and mothers about the place, and attendants of delivery during their last pregnancy in the Kembata Tembaro zone, 2020.

Variables (*n* = 607)		Rural	Urban	Total
		*N* (%)	*N* (%)	*N* (%)
Final decision-maker about place of delivery	My self	181 (43.5)	75 (39.3)	256 (42.2)
	My husband	65 (15.6)	46 (24.1)	111 (18.3)
	Both of us	147 (35.3)	60 ( (31.4)	207 (34.1)
	Others	23 (5.5)	10 (5.2)	33 (5.4)

Preference of your mother About place delivery	Home delivery	305 (73.3)	42 (22)	347 (57.2)
	Institutional delivery	91 (21.9)	135 (70.7)	226 (37.2)
	I don't know	20 (4.8)	14 (7.3)	34 (5.6)

Preference of your mother About attendant of delivery	SBAs	133 (31.9)	121 (63.4)	254 (41.8)
	TBAs	247 (59.4)	32 (16.8)	279 (46)
	Relatives	20 (4.8)	26 (13.6)	46 (7.6)
	Others	16 (3.8)	12 (6.3)	28 (4.6)

Preference of your husband About place of delivery	Home delivery	287 (69)	157 ( (82.2)	444 (73.1)
	Institutional delivery	98 (23.6)	24 (12.6)	122 (20.1)
	I don't know	31 (7.5)	10 (5.2)	41 (6.8)

Preference of your husband About attendant of delivery	SBA	201 (48.3)	135 ( (70.7)	336 (55.3)
	TBAs	167 (40.1)	32 (16.8)	199 (32.8)
	Relatives	30 (7.2)	21 (11)	51 (8.4)
	Others	18 (4.3)	3 (1.6)	21 (3.5)

**Table 5:** Multivariable logistic regression analysis of factors associated with the utilization skilled birth attendance among mothers who gave birth in the past 12 months in the Kembata Tembaro zone, Southern Ethiopia, 2020.

Variables (*n* = 607)		Not utilized	Utilized	Cor	AOR (95% CI)
Name	Category	*N* (%)	*N* (%)		
Place of residence	Urban	37 (19.4%)	154 (80.6%)	1	1
	Rural	215 (51.7%)	201 (48.3%)	0.23	**0.33** (0.22,0.58)^*∗*^

ANC follow-up	≥4 times	147 (66.1%)	45 (33.9%)	1	1
	<4 times	77 (30.4%)	156 (69.6%)	6.62	**4.62** (3.12,7.32)^*∗*^

Overall knowledge on obstetric complications	No	160 (35.5%)	132 (64.5%)	1	1
	Yes	101 (27.4%)	214 (72.6%)	2.57	**3.10** (1.37,5.21)^*∗*^

Occupational status of mother	Housewife	224 (60.2%)	148 (39.8%)	1	1
	Government employee	22 (32.8%)	45 (67.2%)	3.09	**3.35** (1.79,6.27)^*∗*^
	Merchant	85 (50.6%)	83 (49.4%)	1.48	**1.69** (1.70,5.99)^*∗*^

Occupational status of husband	Farmer	220 (62.3%)	133 (37.7%)	-	1
	Government employee	22 (30.1%)	51 (69.9%)	3.83	**3.15** (1.79,6.27)^*∗*^
	Merchant	87 (56.1%)	68 (43.9%)	1.29	**2.69** (1.79,7.09)^*∗*^
	Daily laborer	12 (46.2%)	14 (53.8%)	1.93	0.33 (0.14,0.81)

Number of family members	One	7 (53.8%)	6 (46.2%)	1.01	1.44 (0.26,2.74)
	Two	15 (46.9%)	17 (53.1%)	1.34	1.21 (0.17,4.18)
	Three	47 (40.9%)	68 (59.1%)	1.71	2.05 (0.08,1.92)
	Four	99 (75.6%)	32 (24.4%)	0.38	1.51 (0.76, 2.99)
	More than four	171 (54.1%)	145 (45.9%)	1	1

Age at interview	15–24	81 (58.7%)	57 (41.3%)	1	1
	25–34	175 (44.3%)	220 (55.7%)	1.79	**3.41** (1.57,5.45)^*∗*^
	35 and above	42 (56.8%)	32 (43.2%)	1.08	0.88 (0.37,2.10)

Educational status of mother	Unable to read and write	16 (59.3%)	11 (40.7%)	1	1
	Read and write	114 (41.6%)	160 (58.4%)	2.04	2.76 (0.41,4.81)
	Secondary and above	100 (34.6%)	206 (65.4%)	3.00	**1.50** (1.34,4.19)^*∗*^

Educational status of husband	Unable to read and write	14 (63.6%)	8 (36.4%)	1	1
	Read and write	103 (47.5%)	114 (52.5%)	1.94	1.45 (0.21,3.81)
	Secondary and above	171 (46.5%)	197 (53.5%)	2.02	2.17 (0.54,4.19)

Final decision-maker about place of delivery	Myself	154 (60.2%)	102 (39.8%)	1	1
	My husband	58 (52.3%)	53 (47.7%)	1.38	3.33 (0.79,2.27)
	Both of us	77 (37.2%)	130 (62.8%)	2.55	**3.64** (1.70,7.99)^*∗*^
	Others	16 (48.5%)	17 (51.5%)	1.60	0.33 (0.14, 0.81)

History of still birth	No	367 (66.2%)	187 (33.8%)	1	1
	Yes	16 (30.2%)	37 (69.8%)	4.54	**3.85** (2.14,6.91)^*∗*^

Parity	1	72 (47.7%)	79 (52.3%)	1	1
	2–4	121 (60.2%)	80 (39.8%)	0.60	0.35 (0.29,1.67)
	≥5	127 (49.8%)	128 (50.2%)	0.92	0.69 (0.20,2.79)

Time taken to nearby health facility	≤30 minute	119 (39.9%)	179 (60.1%)	2.43	3.85 (0.14,2.91)
	>30 minute	191 (61.8%)	118 (38.2%)	1	1

Experience on adverse Pregnancy outcome	No	265 (53.1%)	234 (46.9%)	1	**1**
	Yes	58 (53.3%)	50 (46.3%)	0.98	0.72 (0.43, 2.82)

*Note.* ^*∗*^Statistically significant at *p* < 0.05; Hosmer and Lemeshow test, *p*=0.407; the model was adequately fit the data.

## Data Availability

Data can be available upon reasonable request to the corresponding author.
